# RXRα ligand Z-10 induces PML-RARα cleavage and APL cell apoptosis through disrupting PML-RARα/RXRα complex in a cAMP-independent manner

**DOI:** 10.18632/oncotarget.14812

**Published:** 2017-01-25

**Authors:** Lin Xu, Zhiping Zeng, Weidong Zhang, Gaoang Ren, Xiaobin Ling, Fengyu Huang, Peizhen Xie, Ying Su, Xiao-kun Zhang, Hu Zhou

**Affiliations:** ^1^ School of Pharmaceutical Sciences, Fujian Provincial Key Laboratory of Innovative Drug Target Research, Xiamen University, Xiamen, Fujian, China; ^2^ Cancer Center, Sanford Burnham Prebys Medical Discovery Institute, La Jolla, California, USA

**Keywords:** Z-10, RXRα, PML-RARα, interaction, cleavage

## Abstract

The major oncogenic driver of acute promyelocytic leukemia (APL) is the fusion protein PML-RARα originated from the chromosomal translocation t(15;17). All-*trans* retinoic acid (ATRA) and arsenic trioxide cure most patients by directly targeting PML-RARα. However, major issues including the resistance of ATRA and arsenic therapy still remain in APL clinical management. Here we showed that compound Z-10, a nitro-ligand of retinoid X receptor α (RXRα), strongly promoted the cAMP-independent apoptosis of both ATRA- sensitive and resistant NB4 cells via the induction of caspase-mediated PML-RARα degradation. RXRα was vital for the stability of both PML-RARα and RARα likely through the interactions. The binding of Z-10 to RXRα dramatically inhibited the interaction of RXRα with PML-RARα but not with RARα, leading to Z-10's selective induction of PML-RARα but not RARα degradation. Z-36 and Z-38, two derivatives of Z-10, had improved potency of inducing PML-RARα reduction and NB4 cell apoptosis. Hence, RXRα ligand Z-10 and its derivatives could target both ATRA- sensitive and resistant APL cells through their distinct acting mechanism, and are potential drug leads for APL treatment.

## INTRODUCTION

Acute promyelocytic leukemia (APL) originates from the specific chromosomal translocations mostly between chromosomes 15 and 17, leading to the occurrence of the fused oncogene promyelocytic leukemia - retinoic acid receptor-α (PML-RARα) [1]. The corresponding chimeric protein PML-RARα is most often the only driving factor of APL initiation [2, 3]. PML-RARα binds to RARα cognate DNA elements and inhibits RARα-mediated transcription of differentiation genes, leading to the differentiation block of promyelocytes [2, 4]. PML is the organizer of a nuclear structure known as the PML nuclear bodies (NBs), which is important for maintaining cellular homeostasis [5, 6]. Another pathogenic activity of PML-RARα is to disrupt the PML nuclear bodies through its interaction with PML [7].

All-*trans*-retinoic acid (ATRA) induces the transcriptional activity of PML-RARα, leading to the induction of RARα-targeted genes and the differentiation of promyelocyte [2]. ATRA also triggers proteasome- and caspase-mediated degradation of PML-RARα to contribute its efficacy [8–10]. However, ATRA at pharmacological concentration strongly stimulates the transactivation and degradation of RAR, resulting in some RAR-related adverse effects [11, 12]. Also, long term exposure of ATRA often results in the relapse likely due to the mutated RARα moiety in PML-RARα [13, 14]. Arsenic trioxide binds to the PML moiety, leading to SUMO-dependent and ubiquitin-mediated degradation of PML-RARα [15, 16]. The reduction of PML-RARα sensitizes APL cells to the apoptotic signals triggered by arsenic [17]. Because arsenic targets to the PML moiety, it can overcome ATRA-resistance in some cases [13]. However, APL driven by the PLZF-RARα and PML-RARα with point mutations in the PML moiety such as A216V are resistant to arsenic [18, 19]. Thus, novel drugs with different therapeutic mechanisms are desperately needed to overcome the resistance and adverse effects of ATRA and/or arsenic.

Retinoid X receptor-α (RXRα) is a unique nuclear receptor due to its ability to form heterodimers with many other nuclear receptors [20]. It has been elucidated that PML-RARα forms homodimer mediated by the PML moiety, and RXRα binds to PML-RARα through the RARα moiety to form heterotetramer [21]. RXRα is required for the sumoylation of PML-RARα and PML RARα efficient binding to DNA, both of which are necessary for PML-RARα-mediated transformation [22–25]. Several RXRα ligands such as SR11237 and BMS749 trigger the differentiation and apoptosis of APL cells, but they have to work together with cAMP for efficient action [26, 27], leading to the additional side effects from cAMP. Thus, whether some RXRα ligands alone could show striking anti-APL effect is worthy of investigation.

We recently reported that Z-10, a nitro-ligand of RXRα, binds to RXRα with a distinct binding mode and induces a unique conformational change of RXRα [28]. In the current study, we report that Z-10 induced PML-RARα degradation and the apoptosis of both ATRA- sensitive and resistant APL cells in a cAMP-independent manner.

## RESULTS

### Z-10 induces NB4 cell apoptosis

**Figure 1:**
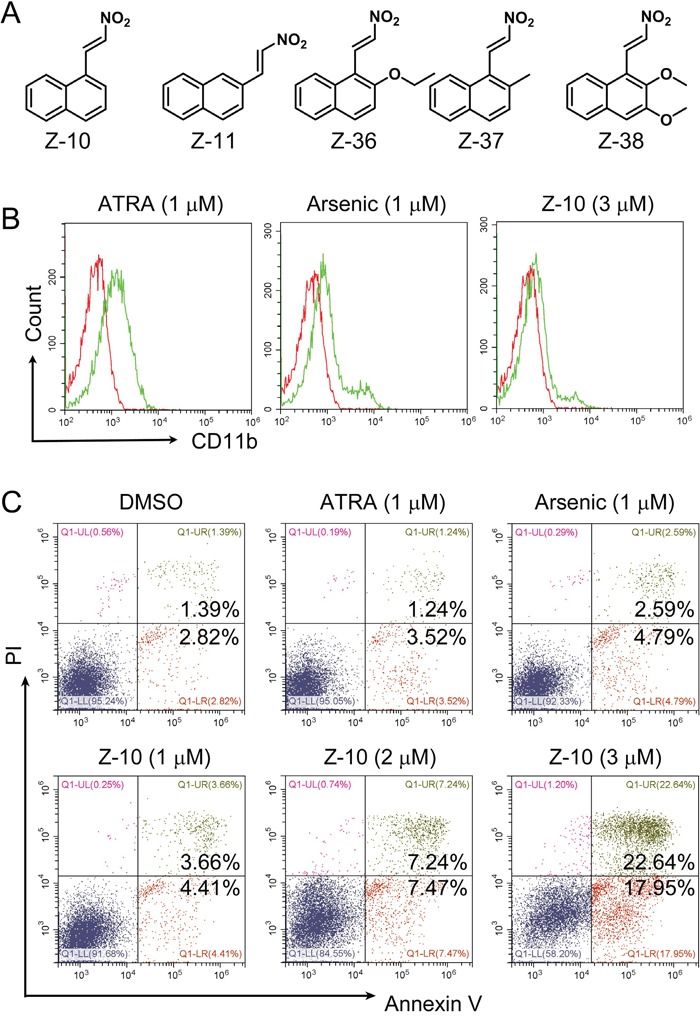
NB4 cell apoptosis induction by Z-10 **A**. The structures of Z serial compounds. **B**. NB4 cells were incubated with ATRA (1 μM), arsenic (1 μM) or Z-10 (3 μM) for two days, and CD11b-positive cells were counted by flow cytometry. Cells treated with DMSO were plotted in red, and cells treated with compounds were plotted in green. **C**. Detection of apoptotic cells by Annexin V-FITC and Propidium iodide (PI) double staining in NB4 cells after incubated with ATRA, arsenic or Z-10 at the indicated concentrations for 36 hours.

RXRα ligands together with cAMP have shown striking anti-APL activities [26, 27]. Recently, we unraveled that Z-10 is a RXRα nitro-ligand with strong growth inhibition and apoptotic induction of MCF-7 breast cancer cells [28]. We therefore explored the activities of Z-10 on the differentiation and apoptosis induction of NB4 cells, a human APL cell line with PML-RARα oncogene [29] (Figure 1). Differentiation induction of Z-10 on NB4 cells was examined by flow cytometry following CD11b the surface differentiation marker staining. As previously reported, ATRA at 1 μM concentration significantly triggered NB4 cell differentiation, as shown by the increased CD11b staining (0.65% CD11b positive cells at DMSO treatment to 30.35% at ATRA treatment) (Figure 1B and Supplementary Figure 1). In comparison, Z-10 at 1 and 2 μM concentrations only had, if there was, minor effect (Figure 5A and Supplementary Figure 3). Z-10 at 3 μM and arsenic at 1 μM had much weaker effect than ATRA at 1 μM on differentiation induction of NB4 cells (Figure 1B and 5A and Supplementary Figure 1 and 3). The apoptotic effect of Z-10 on NB4 cells was examined through Annexin V-FITC/PI double staining assay. Both Z-10 and arsenic at 1 μM significantly promoted NB4 cell apoptosis comparing with ATRA (8.07%, 7.38% and 4.76% NB4 cells underwent apoptosis in the presence of Z-10, arsenic, and ATRA, respectively) (Figure 1C). In addition, the apoptotic induction of Z-10 was in a dose-dependent manner (Figure 1C). These results suggested that Z-10 was of potent capability on inducing NB4 cell apoptosis.

### Z-10 destabilizes PML-RARα

**Figure 2:**
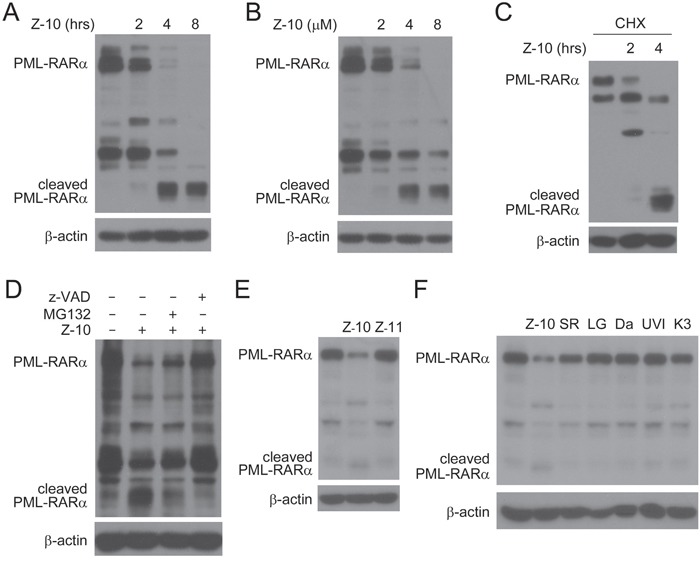
PML-RARα reduction induced by Z-10 through cleavage **A-B**. NB4 cells were treated with 5 μM of Z-10 for the indicated time (A) or treated for 4 hours with the indicated concentrations (B), and PML-RARα expression was analyzed by western blot using anti-RARα antibody. The expression of β-actin was used as a loading control. **C**. NB4 cells were treated with 50 μg/mL cycloheximide (CHX) for 4 hours and 5 μM Z-10 for 2 or 4 hours. Protein expression was analyzed by western blot. **D**. NB4 cells were treated with 5 μM Z-10 together with 20 μM MG132 or 20 μM z-VAD-FMK (z-VAD) for 4 hours, and protein expression was examined by western blot. **E-F**. NB4 cells were treated with 5 μM compounds as indicated for 4 hours, and PML-RARα protein was detected by western blot. SR, LG, Da, UVI and K3 represent SR11237, LG100745, Danthron, UVI3003 and K-8003, respectively.

Through the induction of PML-RARα degradation, arsenic promotes the apoptosis of APL cells [17]. Thus, we explored whether Z-10 affected the stability of PML-RARα. Figure 2A and 2B showed that Z-10 time- and dose- dependently induced PML-RARα reduction in the absence of cAMP. In the presence of cycloheximide (CHX), Z-10 still induced PML-RARα reduction in a time-dependent manner, indicating that Z-10 destabilized PML-RARα protein (Figure 2C). The small fragments of PML-RARα induced by Z-10 suggested that Z-10 destabilized PML-RARα through cleavage (Figure 2A-2C). Z-10-induced PML-RARα reduction and cleavage were strongly inhibited by z-VAD-FMK but only slightly prevented by MG132, implying that caspases but not proteasome was mainly responsible for the degradation and cleavage of PML-RARα (Figure 2D). Z-11 (Figure 1A), an isomer of Z-10, also binds to RXRα but induces a distinct RXRα conformation [28]. Different from Z-10, Z-11 failed to trigger PML-RARα reduction (Figure 2E), indicating the selectivity of RXRα nitro-ligands. Additionally, the strong ability of Z-10 on inducing PML-RARα reduction was unique among RXRα ligands examined (Figure 2F). Therefore, results from above study showed that Z-10 down-regulated PML-RARα mainly through caspase-mediated cleavage in a cAMP independent manner, which might trigger the apoptosis of NB4 cells. This may also explain why Z-10 induced dramatic apoptosis in NB4 cells but not in THP1 cells that do not possess PML-RARα oncoprotein (Supplementary Figure 2).

### Z-10 inhibits the interaction of PML-RARα and RXRα

**Figure 3:**
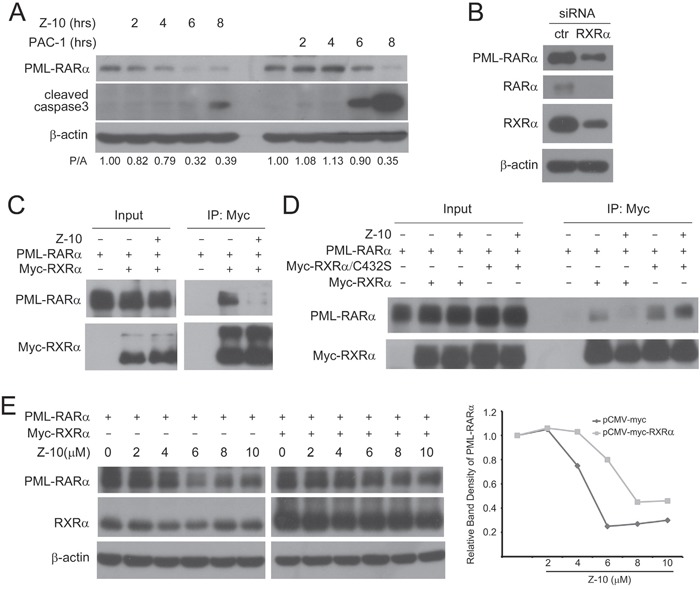
The inhibition of PML-RARα/RXRα complex formation by Z-10 **A**. NB4 cells were treated with 5 μM Z-10 or 50 μM PAC-1 for the indicated time, and the expression of PML-RARα and cleaved caspase3 was analyzed by western blot. Relative protein band densities after normalization to β-actin signal were shown at the bottom of the figure (P/A represents PML-RARα/β-actin). **B**. NB4 cells were infected with sh RXRα or sh control retrovirus for three days. Protein expression levels were analyzed by western blot. **C-D**. Cos-7 cells were transfected with the indicated plasmids for 24 hours and then treated with 5 μM of Z-10 for 4 hours. The complex formations were examined by co-immunoprecipitation and western blot using the indicated antibodies. **E**. Cos-7 cells were transfected with PML-RARα expression plasmid alone or together with Myc-RXRα plasmid for 24 hours, and treated with Z-10 at the indicated concentrations for 4 hours. Protein expression levels were analyzed by western blot. Relative PML-RARα band densities after normalization to β-actin were plotted.

**Figure 4:**
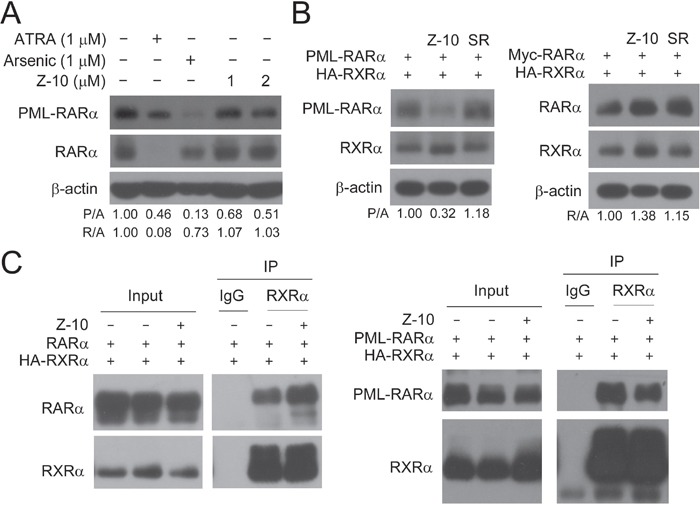
Z-10 had no effect on the stability of RARα **A**. NB4 cells were incubated with Z-10, arsenic or ATRA for 48 hours. The PML-RARα and RARα protein was detected by anti-RARα antibody with β-actin as a loading control. Relative protein band densities after normalization to β-actin signal were shown at the bottom of the figure (P/A represents PML-RARα/β-actin, and R/A represents RARα/β-actin). **B**. Cos-7 cells were transfected with the indicated plasmids for 24 hours, and then treated with 5 μM of Z-10 or SR11237 (SR) for 6 hours. Protein expression levels were analyzed by western blot. **C**. Cos-7 cells were transfected with the indicated plasmids for 24 hours and then treated with 5 μM of Z-10 for 4 hours. The complex formations were examined by co-immunoprecipitation using the indicated antibodies.

We evaluated whether Z-10 activated caspases to cleave PML-RARα. Caspase activator PAC-1 could induce PML-RARα degradation only when caspase3 was highly activated, showing from the simultaneous events of PML-RARα decreased and cleaved caspase3 increased expressions. However, PML-RARα degradation induced by Z-10 was an earlier event before the weak activation of caspase3 (Figure 3A). This suggested that Z-10-induced PML-RARα degradation may not, at least not completely, depend on caspase activation. The binding of RXRα to PML-RARα has been implicated in the development of APL [2]. We then examined the possibility of RXRα regulating PML-RARα stability. When NB4 cells were transfected with siRNA to reduce RXRα expression, the expression levels of both PML-RARα and RARα were accordingly reduced (Figure 3B), suggesting that RXRα binding stabilizes PML-RARα and RARα. We hypothesized that Z-10 could bind to RXRα and inhibit the interaction of RXRα and PML-RARα, thereby leading to the instability of PML-RARα. Our co immunoprecipitation assay showed that PML-RARα and RXRα overexpressed in Cos-7 cells formed strong complex, which was largely disrupted by Z-10 (Figure 3C-3D and 4C). It has been reported that Z-10 fails to bind to RXRα/C432S mutant with cysteine 432 substituted with serine [28]. We found that Z-10 also failed to inhibit the interaction of PML-RARα and RXRα/C432S (Figure 3D), which indicated that Z-10 inhibiting RXRα and PML/RARα interaction relied on its binding to RXRα. In Cos-7 cells, overexpressed RXRα prevented Z-10 induced PML-RARα degradation to a certain extent (Figure 3E). Therefore, RXRα binding could stabilize PML-RARα, and the disruption of the RXRα/PML RARα complex by Z-10 resulted in the instability of PML-RARα. Our data also suggested that the basal activity of caspases was able to induce PML-RARα cleavage once the PML-RARα/RXRα complex was disrupted by Z-10.

### Z-10 has no effect on RARα stability

Because ATRA could bind to both PML-RARα and RARα, one unavoidable side effect of ATRA originates from its strong induction of RARα activation and degradation when patients are treated with ATRA at pharmacologic concentrations [30, 31]. Consistently, we found that ATRA at 1 μM potently induced RARα degradation in NB4 cells (Figure 4A). However, Z-10 only induced PML-RARα degradation and had no apparent effect on the expression level of RARα in NB4 cells (Figure 4A). Similarly, Z-10 dramatically promoted the degradation of overexpressed PML-RARα but not RARα in Cos-7 cells (Figure 4B). As expected, Z-10 failed to inhibit the interaction of RARα and RXRα as did to the interaction of PML RARα and RXRα (Figure 4C). Thus, Z-10 induction of PML-RARα but not RARα degradation was due to its selective inhibition of RXRα interaction with PML-RARα but not with RARα.

### Z-10 induces PML-RARα degradation and apoptosis in ATRA-resistant NB4 cells

**Figure 5:**
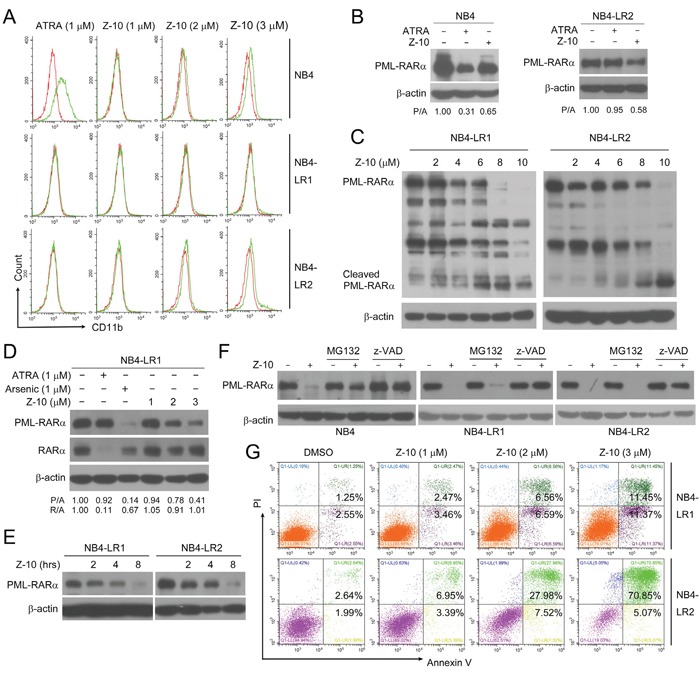
The induction of PML-RARα degradation and apoptosis by Z-10 in ATRA-resistant NB4 cells **A**. NB4, NB4-LR1 or NB4-LR2 cells were incubated with Z-10 or ATRA for two days and CD11b-positive cells were counted by flow cytometry. Cells treated with DMSO were plotted in red, and cells treated with compounds were plotted in green. **B**. NB4 and NB4-LR2 cells were incubated with 1 μM of Z-10 or ATRA for 48 hours. The PML-RARα protein was detected by anti-RARα antibody with β-actin as loading control. **C**. NB4-LR1 and NB4-LR2 cells were treated with Z-10 at the indicated concentration for 4 hours followed by western blot to examine protein expression. **D**. NB4-LR1 cells were incubated with Z-10, ATRA or arsenic at the indicated concentration for 48 hours. The PML RARα and RARα protein was detected by western blot using anti-RARα antibody. Relative protein band densities after normalization to β-actin signal were shown at the bottom of the figure (P/A represents PML-RARα/β-actin, and R/A represents RARα/β-actin). **E**. Western blot analysis of PML-RARα expression in NB4-LR1 and NB4-LR2 cells treated with 5 μM of Z-10 for the indicated hours. **F**. NB4, NB4-LR1 and NB4-LR2 cells were treated with 5 μM Z-10 together with 20 μM MG132 or 20 μM z-VAD-FMK (z-VAD) for 4 hours, and protein expression was examined by western blot. **G**. NB4-LR1 and NB4-LR2 cells were incubated with Z-10 at the indicated concentrations for 36 hours, and the detection of apoptotic cells was carried out by Annexin V-FITC/PI double staining.

ATRA is a widely used and effective clinical drug for APL [32, 33]. However, a fraction of APL is refractory to ATRA due to long-term ATRA treatment and PML-RARα mutations [13, 14]. As previously reported, ATRA could strongly induce the differentiation of NB4 cells but not NB4-LR1 and NB4-LR2 cells, two ATRA-resistant cell lines [14, 34] (Figure 5A and Supplementary Figure 3). Consistently, ATRA promoted PML-RARα degradation only in NB4 cells but not in NB4-LR1 or NB4-LR2 cells (Figure 5B and 5D). However, Z-10 was able to induce PML-RARα degradation in all the three cell lines (Figure 5B-5E). Also, Z-10 dose- and time-dependently induced PML-RARα reduction and cleavage in both NB4-LR1 and NB4-LR2 cell lines mainly through caspase-mediated pathway (Figure 5C-5F). Similarly, Z-10 did not affect the expression of RARα in NB4-LR1 cells (Figure 5D). Although Z-10 did not show apparent differentiation induction of NB4-LR1 and NB4-LR2 cells (Figure 5A and Supplementary Figure 3), it significantly promoted the apoptosis of these two cell lines in a dose-dependent manner (Figure 5G). Thus, Z-10 could induce PML-RARα degradation and apoptosis in ATRA-resistant NB4 cells.

### Z-36 and Z-38 are optimized Z-10 derivatives

**Figure 6:**
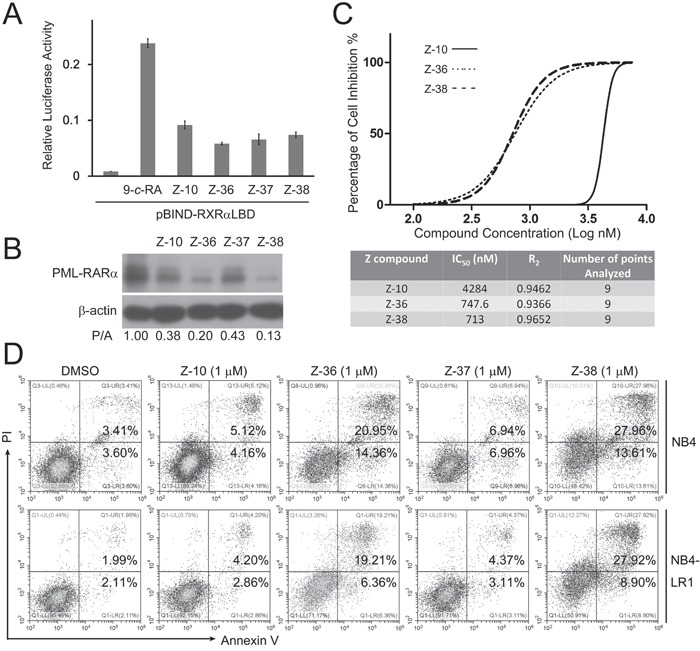
Z-36 and Z-38 were optimized Z-10 derivatives **A**. HEK293T cells were transfected with pBIND-RXRα-LBD and pG5-luc for 24 hours, and treated with 0.1 μM 9-*cis*-RA (9-*c*-RA) and the indicated compounds (5 μM). Luciferase activities were measured 12 hours after treatment and relative luciferase activity was plotted. **B**. NB4 cells were treated with 5μM of the indicated compounds for 4 hours. The PML-RARα protein was detected by anti-RARα antibody with β-actin as loading control. **C**. NB4 cells were plated at a density of 1 × 10^5^ cells/ml in a 96-well plate. Cells were treated with Z-10 at increased concentrations (0.1, 0.5, 1, 2, 3, 4, 5, 6, and 7.5 μM) for 36 hours, and cell proliferation was measured by MTS assay. Data were analyzed and plotted using GraphPad Prism software. **D**. NB4 and NB4-LR1 cells were incubated with 1 μM of compounds as indicated for 36 hours, and the detection of apoptotic cells was carried out by Annexin V-FITC/PI double staining.

To improve the potency of Z-10, we synthesized a series of Z-10 derivatives and examined their effects in NB4 cells. Among the synthesized Z-10 derivatives examined, we found that Z-37 had similar ability as Z-10 while Z-36 and Z-38 possessed stronger ability than Z-10 on inducing PML-RARα degradation (Figure 1A and Figure 6B). In consistent with their ability on degrading PML-RARα, Z-36 and Z-38 showed stronger potency on inhibiting NB4 cell viability (The IC_50_ of Z-10, Z-36 and Z-38 were 4.284, 0.7476 and 0.713 μM, respectively). Z-36 and Z-38 also displayed stronger apoptosis induction of NB4 and NB4-LR1 cells than Z-10 (Figure 6D). Z-10 and its derivatives exhibited similar stimulating effect of RXRα transactivation (Figure 6A), suggesting that their anti-APL ability was not directly correlated to their activation of RXRα transactivation.

## DISCUSSION

Although ATRA and arsenic have improved the overall survival rate of APL patients [35, 36], there exist major issues in the APL treatment such as ATRA and arsenic resistance as well as their off target side effects [18, 19, 37]. In our current study, we provided a potential drug lead that may overcome ATRA-resistance and ATRA-related side effects through its distinct acting mode.

Z-10, as the first identified nitro-ligand of RXRα, has distinct properties from classic RXRα ligands in both molecular structures and RXRα-dependent activities [28]. cAMP-dependent and PKA-catalyzed phosphorylation of PML-RARα is crucial for some RXRα ligands to efficiently induce PML-RARα degradation and APL cell apoptosis [38, 39]. Here, we showed that Z-10 is unique among RXRα ligands in that it has strong anti-APL activity in the absence of cAMP (Figure 1 and Figure 2). This should, at least in part, be attributed to its induction of a distinct RXRα conformation, resulting in the dissociation of PML-RARα from RXRα (Figure 3C-3D and Figure 4C). The cAMP-independent PML-RARα degradation by Z-10 also suggested that Z-10 may represent a promising drug lead in a distinct class of RXRα ligands for APL treatment.

RXRα forms strong heterotetramers with PML-RARα, and disruption of their interaction inhibits APL initiation and development [24, 25]. RXRα has been shown to be required for PML-RARα sumoylation and efficiently binding to DNA, both of which are essential events for APL transformation [22, 23]. In our research, we unraveled that RXRα was also vital for the stability of PML-RARα (Figure 3), which provided a new mechanism underlying RXRα actions in APL. It is conceivable that this contribution of RXRα also originates from its interaction with PML-RARα. Therefore, a feasible approach to eliminate PML-RARα is to abrogate its interaction with RXRα. Indeed, Z-10 could potently inhibit the interaction of PML-RARα and RXRα (Figure 3C-3D and Figure 4C), leading to the caspase-mediated cleavage of PML-RARα (Figure 2D). We hypothesized that the binding of RXRα may preclude the cleavage of PML-RARα through masking the cleavage sites or inhibiting the access of caspases. Figure 3A indicated that PML-RARα reduction induced by Z-10 preceded caspase activation, implying that basal activity of caspases was enough for catalyzing PML-RARα cleavage once the PML-RARα/RXRα complex was disrupted.

Different from the heterodimer formed by RARα and RXRα, PML-RARα and RXRα forms heterotetramer. In the heterotetramer, PML-RARα not only interacts with RXRα through RARα moiety, but also interacts with each other through PML moiety [23, 25]. These may underlie the different modes of RXRα interaction with PML-RARα and RARα. It is conceivable that the conformational change induced by Z-10 may only affect RXRα interaction with PML-RARα but not with RARα, a possibility that was confirmed by our co immunoprecipitation assay (Figure 4C). Consistently, even though RXRα was vital for the stability of both PML-RARα and RARα, Z-10 binding to RXRα selectively reduced the stability of PML-RARα (Figure 4A-4B and Figure 5D). This property might allow Z-10 to circumvent some of ATRA's side effects. RXRα could form heterodimers with many other nuclear receptors [40]. If Z-10 did not affect RXRα binding to RARα, it likely has no or only minor effect on RXRα heterodimerization with other nuclear receptors.

One limitation in APL treatment is ATRA resistance [14, 41]. Due to the mutations of the RARα moiety that directly affect the binding of ATRA, it should be difficult to solve this issue through modifying ATRA or using other RARα ligands. Since Z-10 inhibited PML RARα through binding to RXRα, it may overcome this problem. Indeed, our results showed that Z-10 induced PML-RARα degradation and apoptosis in ATRA-resistant NB4 cells (Figure 5). This implied that RXRα was also important to maintain the stability of the mutated PML RARα, and Z-10 was able to inhibit the interactions of RXRα with the mutated PML-RARα. It remains to be investigated whether Z-10 could also overcome the arsenic-resistance.

Z-36 and Z-38 are two derivatives of Z-10 only after simple modifications (Figure 1A). However, these two derivatives had substantially improved potency (Figure 6B-6D), which suggested that Z-10 can be further optimized in term of APL treatment. Animal studies of Z-10 and its derivatives will be needed to confirm their efficacy and study their potential side effects. It will also be interesting to study the synergistic effect of Z-10 and ATRA in animal experiments.

## MATERIALS AND METHODS

### Reagents and antibodies

Antibodies for RARα (sc-551), RXRα (sc-553), PARP-1/2 (sc-7150), c-Myc (sc-40) were purchased from Santa Cruz Biotechnology; Antibody for β-actin (A2228), ATRA (R2625), SR11237 (S8951), LG 100754 (SML0771), Danthron (D108103), Arsenic, and 9-*cis*-RA (R4643) were purchased from Sigma-Aldrich; Antibody for human FITC-CD11b was from eBioscience; Antibody for Cleaved caspase-3 (#9661) was from Cell Signaling Technology; PAC-1(Procaspase-activating compound-1) was from MedChem Express; UVI3003 (3303) was from Tocris Bioscience; K-8003 and Z compounds were stored in our laboratory.

### Compound synthesis

General procedure for the synthesis of Nitroolefins (Z-10, Z-11, Z-36, Z-37 and Z-38) was via the Henry reaction as previously described [28].

### Cell culture

APL cell lines NB4 and NB4-derived ATRA-resistant NB4-LR1 and NB4-LR2 were cultured in RPMI 1640 medium (Thermo Fisher Scientific). African green monkey kidney fibroblast-like cell COS-7 and human embryonic kidney cell 293T were cultured in DMEM medium (Thermo Fisher Scientific). The mediums were supplemented with 10% fetal bovine serum (FBS, Gibco), penicillin (100 IU/ mL), and streptomycin (100 μg/mL), and the cells were maintained at a humidified incubator at 37 °C and 5% CO_2_. For experiments, cells were incubated with compounds with 0.01-0.1% DMSO treatment as control.

### Plasmids and transfection

PSG5-PML-RARα plasmid was a gift from Dr. Jun Zhu (Shanghai Jiao-Tong University School of Medicine). Myc-RXRα/C432S was constructed with standard methods. HA-RXRα, Myc-RARα, Myc-RXRα, pBind-RXRα-LBD, pGL6-TA-RXRE, pG5 were preserved in our laboratory. COS-7 cell transfections were carried out by using TurboFect Transfection Reagent (R0531, Fermentas) according to the instructions of the manufacturer. NB4 cell transfections were carried out by using retrovirus infection, and RXRα shRNA sequence is CAAGGACTGCCTGATTGAC.

### Western blot

Cells were lysed, and equal amounts of the lysates were loaded onto 8% sodium dodecylsulfate–polyacrylamide gel, electrophoresed, and transferred to polyvinylidene difluoride membranes (Millipore). The membranes were blocked with 5% skimmed milk in TBST (50 mM Tris-HCl [pH 7.4], 150 mM NaCl, and 0.1% Tween 20) for 1 hour and then incubated with primary antibodies overnight at 4°C, followed by secondary antibodies for 2 hours at room temperature. The detection was performed by the ECL system (Thermo). β-actin was used as an internal control. Protein band density was quantified using software Quantity One-4.6.2.

### Co-immunoprecipitation assay

Cells were harvested and lysed in buffer containing 20 mM Tris-HCl (pH 7.5), 150 mM NaCl, 1 mM EDTA, 1 mM EGTA, 1% Triton X-100, 2.5 mM sodium pyrophosplate, 1 mM β-glycerophosplate and 1 mM Na_3_VO_4_, with proteinase inhibitor cocktail. Immunoprecipitation was performed as previously described [28, 42, 43].

### Apoptosis and differentiation assay

Three-milliliter cultures of NB4, NB4-LR1 and NB4-LR2 cells at a density of 3×10^5^ cells/mL were incubated with compounds and equal amount of DMSO for 36 hours. The apoptosis of the cells was detected using FITC Annexin V Apoptosis Detection Kit I (556547, BD Biosciences) according to the instructions of the manufacturer. Cell surface differentiation antigen CD11b was measured using fluorescein isothiocyanate-labeled antibodies with isotype controls via flow cytometry.

### MTS assay

NB4 cells were seeded in 96-well culture plate at a cell density of 1×10^5^ cells/mL and treated with compounds for 36 hours. The cell viability was evaluated using an MTS assay kit (Promega, G3580). Briefly, MTS (10 mg/ml, 3-(4,5 dimethylthiazol-2-yl)-5-(3-carboxymethoxyphenyl)-2 (4-sulfophenyl)- 2H-tetrazolium) was added to each well followed by incubation for 2 hours at 37 °C. The absorbance was measured at a wavelength of 490 nm using an Elisa-Reader.

### Mammalian one-hybrid assay

293T cells were cotransfected with pG5-luc reporter (Promega) together with the RXRα-LBD fused with the DNA-binding domain of Gal4. After 24 hours, cells were treated with DMSO, Z compounds, or 9-*cis*- RA. After 12 hours, cells were lysed by passive lysis buffer. Firefly and Renilla luciferase activities were quantitated using the Dual-Luciferase Reporter Assay System (Promega, E1960). Transfection and expression efficiency was normalized to Renilla luciferase activity.

### Statistical analysis

Results were derived at least three independent experiments and shown as the mean ± standard deviation. The student*'s t*-test was applied for the statistic analysis, and *p* <0.05 was considered significant.

## SUPPLEMENTARY MATERIALS FIGURES AND TABLES


