# Supplementary material for: RXRα ligand Z-10 induces PML-RARα cleavage and APL cell apoptosis through disrupting PML-RARα/RXRα complex in a cAMP-independent manner
Source: Oncotarget. 2017 Jan 25;8(7):12311–22. doi: 10.18632/oncotarget.14812 (PMC5355346; doi:10.18632/oncotarget.14812)
Supplement: Supplementary file 1 [file oncotarget-08-12311-s001.pdf]

RXRα ligand Z-10 induces PML-RARα cleavage and APL cell apoptosis through disrupting PML-RARα/RXRα complex in a cAMP-independent manner

SUPPLEMENTARY FIGURES

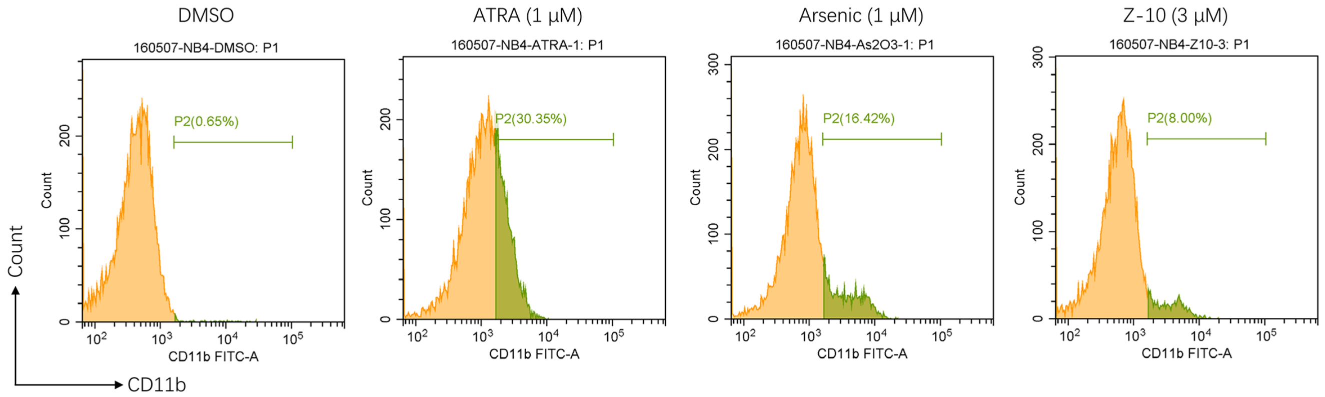

Supplementary Figure 1 (related to Figure 1B): NB4 cells were incubated with DMSO, ATRA (1 µM), arsenic (1 µM) or Z-10 (3 µM) for two days, and CD11b-positive cells were quantitated by flow cytometry.

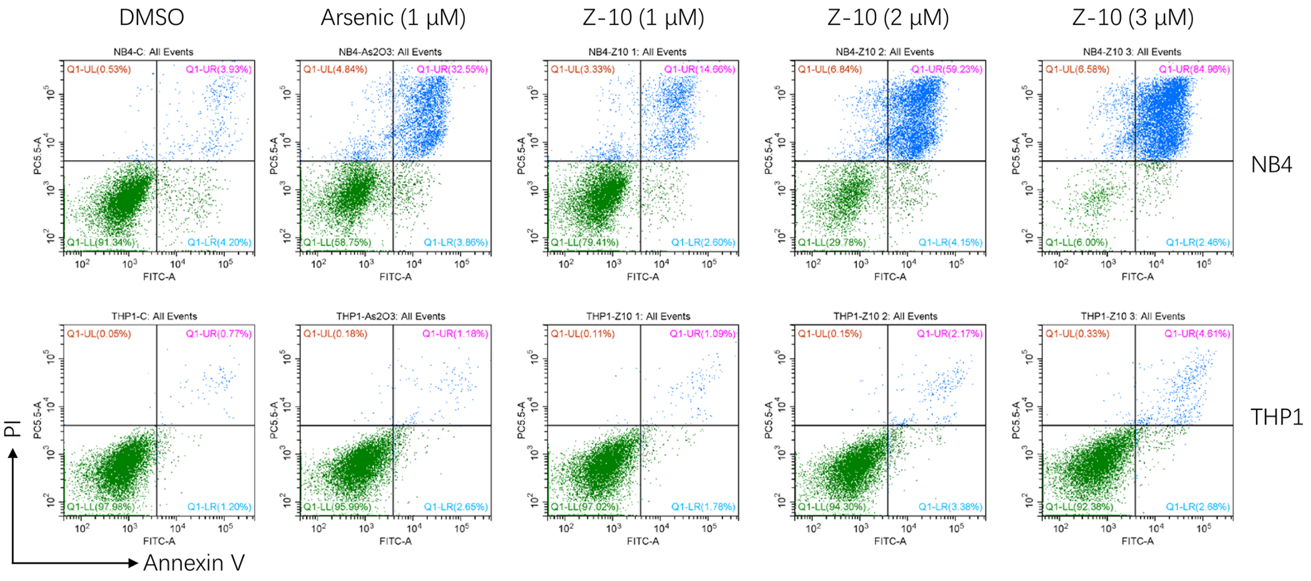

Supplementary Figure 2: NB4 and THP1 cells were plated at a density of 1 × 10^5 cells/ml in a 6-well plate. Cells were treated with arsenic and Z-10 at indicated concentrations for 36 hours, and the detection of apoptotic cells were carried out by Annexin V-FITC/PI double staining.

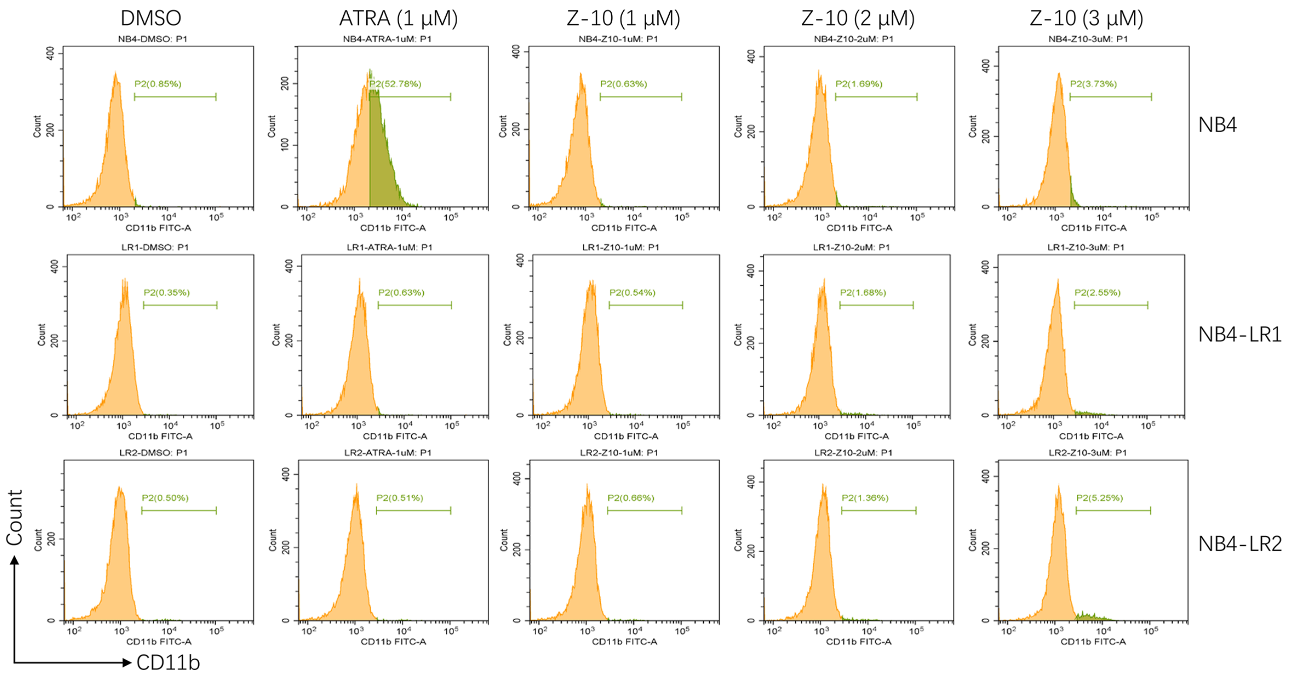

Supplementary Figure 3 (related to Figure 5A): NB4, NB4-LR1 or NB4-LR2 cells were incubated with DMSO, ATRA, Z-10 for two days, and CD11b-positive cells were quantitated by flow cytometry.
